# Effect of Metformin on Meibomian Gland Epithelial Cells: Implications in Aging and Diabetic Dry Eye Disease

**DOI:** 10.3390/life14121682

**Published:** 2024-12-18

**Authors:** Leon Rescher, Swati Singh, Ingrid Zahn, Friedrich Paulsen, Martin Schicht

**Affiliations:** 1 Institute of Functional and Clinical Anatomy, Friedrich-Alexander University Erlangen-Nürnberg, 91054 Erlangen, Germany; leon.rescher@googlemail.com (L.R.); dr.swati888@yahoo.com (S.S.); ingrid.zahn@fau.de (I.Z.); friedrich.paulsen@fau.de (F.P.); 2 Centre for Ocular Regeneration, L.V. Prasad Eye Institute, Hyderabad 500034, India

**Keywords:** hMGECs, meibomian gland epithelial cell, dry eye disease, diabetes, metformin, lipid

## Abstract

Background: Metformin, a commonly prescribed medication for managing diabetes, has garnered increasing interest as a potential therapeutic option for combating cancer and aging. Methods: The current study investigated the effects of metformin treatment on human meibomian gland epithelial cells (hMGECs) at morphological, molecular, and electron microscopy levels. HMGECs were stimulated in vitro with 1 mM, 5 mM, and 10 mM metformin for 24, 48, and 72 h. The assessed outcomes were cell proliferation assays, lipid production, ultrastructural changes, levels of IGF-1, Nrf2, HO-1, apoptosis-inducing factor 1 (AIF1) at the protein level, and the expression of oxidative stress factors (matrix metallopeptidase 9, activating transcription factor 3, CYBB, or NADPH oxidase 2, xanthine dehydrogenase). Results: Morphological studies showed increased lipid production, the differentiation of hMGECs after stimulation with metformin, and the differentiation effects of undifferentiated hMGECs. Proliferation tests showed a reduction in cell proliferation with increasing concentrations over time. AIF1 apoptosis levels were not significantly regulated, but morphologically, the dying cells at a higher concentration of 5-10 mM showed a rupture and permeabilization of the plasma membrane, a swelling of the cytoplasm, and vacuolization after more than 48 h. The IGF-1 ELISA showed an irregular expression, which mostly decreased over time. Only at 72 h and 10 mM did we have a significant increase. Mitochondrial metabolic markers such as Nrf2 significantly increased over time, while HO-1 decreased partially. The RT-PCR showed a significant increase in MMP9, CYBB, XDH, and ATF with increasing time and metformin concentrations, indicating cell stress. Conclusions: Our results using a cell line suggest that metformin affects the cellular physiology of meibomian gland epithelial cells and induces cell stress in a dose- and duration-dependent manner, causing changes in their morphology and ultrastructure.

## 1. Introduction

Metformin is a commonly used drug for treating diabetes mellitus (DM), which reduces glucose levels and improves insulin sensitivity [1,2]. Its application has expanded to other diseases like cancer, and it has been used as an anti-aging treatment [2]. Metformin is consumed in doses of 500 mg twice daily, along with growth hormone, to increase the lifespan [2]. Its antiaging effect is believed to occur by affecting the insulin-like growth factor (IGF) signaling pathway that regulates cell proliferation and differentiation [2]. Diabetes and aging are both risk factors for dry eye disease (DED), especially meibomian gland dysfunction (MGD), and both conditions require the use of metformin [3,4]. DED prevalence is 15 to 53% in DM individuals. A meta-analysis of 48 studies evaluated risk factors for DED and reported DM to have 1.15 times higher odds of DED, although the studies were highly heterogeneous (86.7%) [5]. Of all drugs used to treat DM, metformin is the only drug that has been linked to DED. A case–control study of 120 diabetics and 120 age-matched controls revealed 38.3% of DM to have DED [4]. Interestingly, the authors found that metformin was the independent variable associated with DED in DM patients. It is hypothesized that the anticholinergic effects already discussed by Chew et al. are the reason for the negative effect of metformin [6]. It is known that the anticholinergic burden appears to be associated with DED [7]. The fact that metformin also has a positive effect is shown by the protective effects of metformin on the retina of DM patients, contrary to the DED scenario [8]. Studies have shown that metformin not only has a blood glucose-lowering effect in type 2 diabetes mellitus but also reduces the risk of cardiovascular disease (CVD) by positively influencing risk factors such as obesity, dyslipidemia, and high blood pressure [9].

The pathogenesis of MGD in DM is multimodal, ranging from hyperglycemia to dysregulation of neuronal activity in the cornea or blink reflex [10,11,12,13,14]. Metformin in a streptozotocin-induced DM rat model showed increased anti-oxidant levels and maintained acinar morphology; it required high doses of metformin [11]. The effects of metformin have never been studied before on human meibomian glands, nor in non-diabetic animals or humans (relevant for non-diabetic indications). The current study hypothesizes that metformin improves the function of the meibomian gland in lipid secretion. In the current study, the effects of metformin on the epithelial cells of the human meibomian gland were therefore investigated at the morphological, molecular, and electron microscopic level using an immortalized human meibomian gland epithelial cell line (hMGEC).

## 2. Materials and Methods

### 2.1. Cell Line

HMGEC was obtained from David A. Sullivan (also Schepens in Boston) and was cultured under standard conditions with and without 10% fetal calf serum to initiate differentiation, as described before [15].

HMGECs were stimulated with different metformin concentrations. Cell ultrastructure and viability were studied with transmission electron microscopy (TEM) and proliferation assays. In addition, a proliferation assay and an ELISA study of culture supernatant showed positive and negative effects on proliferating behavior.

### 2.2. HMGEC Cultures and Metformin Stimulation

The immortalized cell line hMGEC was used for experiments that were first established in 2010 [16]. HMGECs were grown in six-well plates (2 mL/well) with 2 × 104 cells/cm^2^ in a proliferation medium. hMGECs were cultured in a proliferative state with serum-free keratinocyte medium supplemented with 5 ng/mL epidermal growth factor and 50 μg/mL bovine pituitary extract (#17005075, Thermo Fisher Scientific, Waltham, MA, USA). After 3 days, the proliferation medium was replaced by a differentiation medium composed of 500 mL Dulbecco’s modified Eagle’s medium and F-12 HAM’s F12 (Biochrom/Merck, Darmstadt, Germany, #FG4815), 10 ng/mL epidermal growth factor (Sigma Aldrich, St. Louis, MO, USA, #E4127), and 10% fetal calf serum—FKS (#10270 Gibco/Life Technologies). Cells were incubated in differentiation medium for 24 h. Culture media and supplements were purchased from Gibco Life Technologies, Karlsruhe, Germany, and Biochrom AG, Berlin, Germany. To stimulate the hMGECs during the proliferating or undifferentiated stage, cells were preincubated for 24 h in a keratinocyte serum-free medium without supplements before being transferred to a medium containing the specific agents. In the differentiating stage, cells were preincubated in a proliferation medium with the appropriate stimulants. After 24 h, the medium was replaced with a differentiation medium containing the same agents. An amount of 250 mM of metformin stock solution was prepared to stimulate the cells with metformin. Metformin was prepared by dissolving one tablet of Siofor 500 (Berlin Chemie AG, Berlin, Germany), which contains 390 mg metformin, in 12,078 mL of distilled water and sterile filtering it for use in cell culture. The stock solution was diluted in a differentiation medium to prepare 1 mM, 5 mM, and 10 mM of metformin. Cells were stimulated for 24, 48, and 72 h for each concentration. Untreated cells served as the control.

### 2.3. Enzyme-Linked Immunosorbent Assay (ELISA)

An ELISA analysis was performed using cell protein to determine the concentration of nuclear factor erythroid 2-related factor-2 (Nrf2, Antibodies-Online, Aachen, Germany A311930), Heme Oxygenase-1 (HO-1 Antibodies-Online, Germany, A313350), insulin-like growth factor 1 (IGF-1, Biozol Diagnostic, Eching, Germany, BOB-EK0376), and apoptosis-inducing factor-1 (AIF1, Antibodies-Online, Germany, A2048) according to the manufacturer’s protocols. The analysis was performed using a microplate spectrophotometer (ELISA reader ClarioStar, BMG Labtech GmbH, Munich, Germany) at wavelengths of 405 nm and 450 nm to measure absorbance. By comparison with the standard series and the determined values for the antigen concentration (protein concentration), each sample was calculated in ng/mg.

### 2.4. Cell Proliferation Assays

To test whether metformin affects the proliferation rate of hMGECs, a BrdU assay (Abcam, Cambridge, UK, ab126556) was used according to the manufacturer’s instructions.

### 2.5. Lipid Production

After stimulation, hMGECs were incubated with 5 µM Lipi-Red Solution (Dojindo Laboratories, Kumamoto, Japan) dissolved in PBS for 30 min at 37 °C according to the manufacturer’s instructions. Lipid proportions in the hMGECs were analyzed using the fluorescence microscope Keyence BZ-X800E (Keyence BZ-X800E, Osaka, Japan).

### 2.6. Transmission Electron Microscopy

hMGECS were fixed with an ITO fixative solution of 25% paraformaldehyde, 25% glutardialdehyde, and 0.1 M cacodylate buffer. They were then prepared for Epon embedding according to the known procedures. Ultrathin sections were prepared with an ultramicrotome (Ultracut Leica, Jena, Germany) and examined with a JEM-1400Plus electron microscope (JEOL, Tokyo, Japan). Morphological changes and the density of lipid bodies and the associated vacuoles were assessed using SightX-Viewer software (Jeol, version 1.2.3.537).

### 2.7. Realtime RT-PCR

For assessing the gene expression of oxidative stress markers via the TaqMan^®^ Gene Expression Assay System, the total RNA of hMGECs treated with different concentrations of metformin was isolated using RNA solve reagent (Omega Bio-TEK, Norcross, GA, USA) according to the manufacturer’s instructions. Afterward, RNA concentrations were quantified with a NanoDrop ND-1000 spectrophotometer (NanoDrop Technologies, Wilmington, DE, USA), and a cDNA synthesis was performed with 2 µg RNA from the respective tissue samples. The reverse transcription was performed with the First Strand cDNA Synthesis Kit (Thermo Fisher Scientific) according to the manufacturer’s recommendations. The quantitative real-time PCR was conducted with a Light Cycler 480 II instrument (Roche) and TaqMan Fast Advanced Master Mix (Ref. 4444557, Thermo Fisher/Applied Biosystems), combined with the corresponding TaqMan Assay (primer/probe) (Thermo Fisher/AppliedBiosystems, Table 1) according to the manufacturer’s instructions (UNG incubation 50 °C 2 min, polymerase activation 95 °C 20 s, PCR 55 cycles, denature 95 °C 3 s, anneal/extend 60 °C 30 s). The raw data were evaluated using the ΔΔC(T) method, as described previously [17,18]. All primers used can be taken from the Table 1 and Table 2.

### 2.8. Statistical Analysis

A statistical analysis was performed using PRISM 10.0 software (GraphPad, La Jolla, CA, USA). A *p* < 0.05 was considered significant. The raw data were normalized relative to the expression of the control (relative to control = 1, above 1 means upregulated, below 1 means downregulated). For all figures, we used a One-Way ANOVA (Kruskal–Wallis with Dunn’s test (uncorrected)).

## 3. Results

### 3.1. Ultrastructural Changes

**Figure 1:**
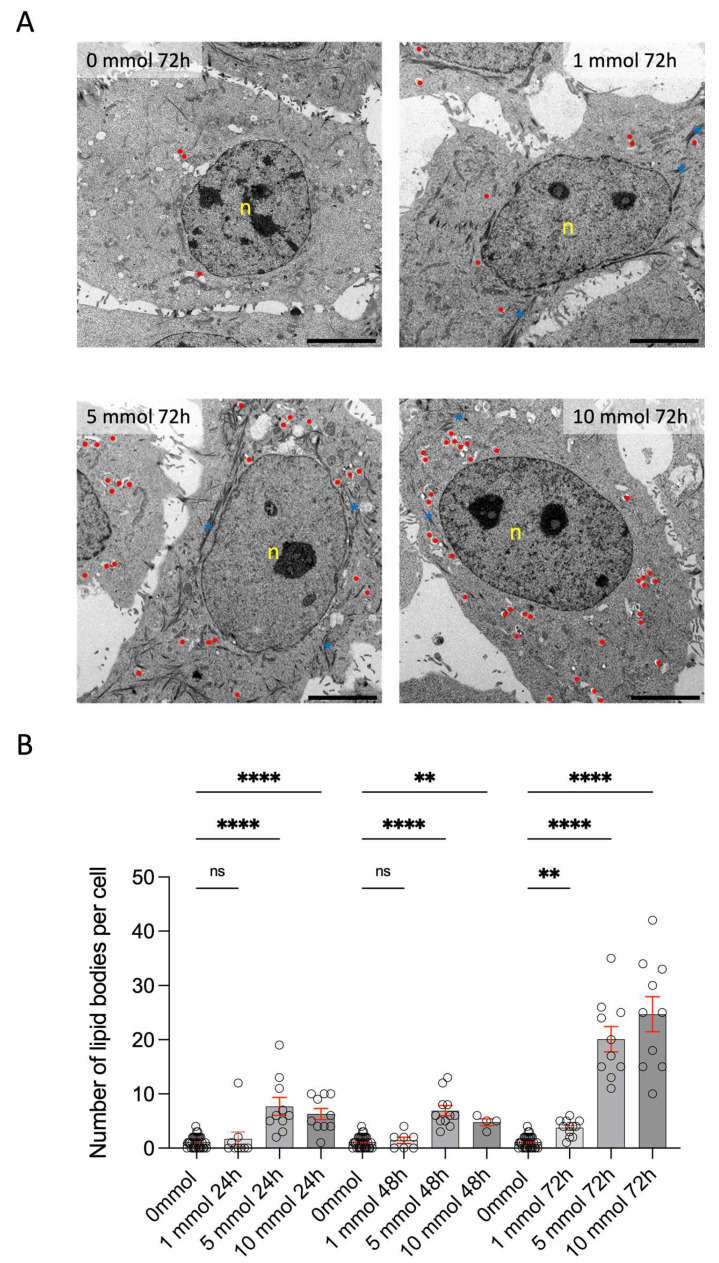
Morphology and lipid bodies. (**A**) Electron micrograph of stimulated hMGECs at different time points and concentrations. Red points = lipid bodies, n = nucleuls, scale bar = 5 μm. (**B**) Quantification of the percentage of lipid bodies at different time points and concentrations. Statistic is the mean + SEM, ns = not significant; ** *p* < 0.01, **** *p* < 0.0001, One-Way ANOVA (Kruskal–Wallis with Dunn’s test (uncorrected)).

TEM studies of differentiated hMGECs showed an increase in lipid vacuole density following 24 h stimulation with all metformin concentrations, i.e., 1 mM, 5 mM, and 10 mM (Figure 1), compared to unstimulated cells. With increasing concentrations of metformin, the epithelial cells were abundant in keratin filament bundles (blue stars), which formed complex cytoskeletal networks crucial for cell type-specific functions. After prolonged exposures for more than 48 h with 10 mM metformin, cells started showing signs of induced cell death (Supplementary Figure S1A) (rupture and permeabilization of the plasma membrane and swelling of the cytoplasm and higher vacuolization); at 72 h with 10 mM metformin, most cells showed low electron density of the cytoplasm, and organelles were completely destroyed (Supplementary Figure S1B)

### 3.2. Lipid Production and Cell Proliferation

**Figure 2:**
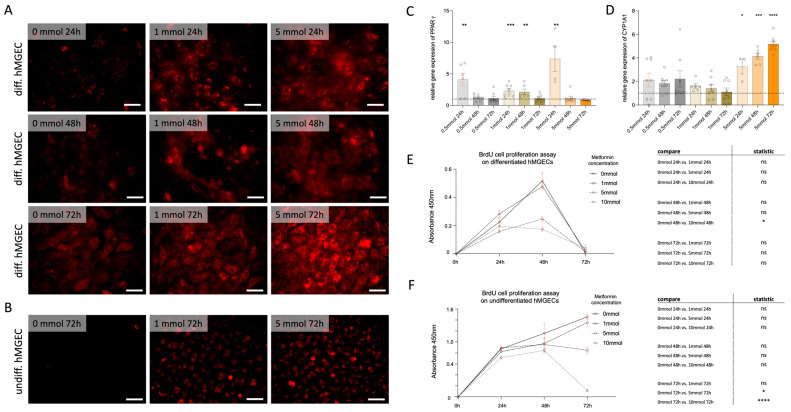
Lipi-Red staining, gene expression of PPARγ and CYP1A1, and cell proliferation assay of stimulated hMGECs. (**A**) Representative images of lipogenesis in differentiated hMGECs after treatment with metformin (0, 1 and 5 mmol) at 24, 48 and 72 h, visualized by Lipi-Red staining. Lipi-Red staining exhibits high selectivity for lipid droplets. An increase in lipid droplets within the cells was still observed up to 72 h. n = 4. Scale bar = 50 µm. (**B**) Differentiation effects of undifferentiated hMGECs with a slight increase in the amount of Lipi-Red staining. n = 4. Scale bar = 50 µm. (**C**,**D**) Diagrams showing the differences in gene expression of PPAR***γ*** and CYP1A1 in hMGEC (n = 4). The data display the mean ± SEM from n = 4 per group. The raw data were normalized relative to the expression of the control (relative to control = 1, above 1 mean upregulated, below 1 means downregulated), * *p* < 0.05, ** *p* < 0.01, *** *p* < 0.001, **** *p* < 0.0001 Kruskal–Wallis with Dunn’s (uncorrected). (**E**,**F**) Proliferation assay of stimulated diff. and undiff. hGMECs at different time points and concentrations. Statistic is the mean + SEM, ns = not significant; * *p* < 0.05, **** *p* < 0.0001, One-Way ANOVA (Kruskal–Wallis with Dunn’s test (uncorrected)).

Lipi-Red staining showed increased lipid production in differentiated hMGECs after metformin exposures of 1 mM and 5 mM for 72 h (Figure 2A). We were also able to demonstrate this effect in lipid counting (Figure 1B). Lipi-Red was most visible after 72 h incubation with 1 mM and 5 mM metformin (Figure 2A). In addition, we determined the gene expression of enzymes such as PPAR***γ*** (peroxisome proliferator activated receptor gamma) and CYP1A1 (Cytochrom-P450 1A1), which catalyze lipid peroxidation and are involved in lipid synthesis. While PPAR***γ*** is significantly upregulated at all concentrations only at the beginning, CYP1A1 shows a significant increase over time after 5 mM metformin. This confirms both the perception in the Lipi-Red staining and the increase in lipid bodies in the TEM analysis.

The differentiated hMGECs are characterized by the fact that they increasingly stop proliferating and instead increase lipid synthesis. The BRDU assay should show that the differentiation processes increase and the cells lose their ability to proliferate. The question arises as to what effect metformin has on the ability to proliferate. After 48 h, differentiated cell proliferation is significantly reduced at higher metformin concentrations of 5 mM and 10 mM compared to the control (Figure 2E). An amount of 1 mM showed no inhibitory effect compared to the control. No proliferation could be detected after 72 h.

Undifferentiated cells showed a significant reduction in proliferation after 24 h of stimulation with 10 mM metformin (Figure 2F). Although not significant, a reduction in proliferation was observed within 48 h after stimulation with 1 mM, 5 mM, or 10 mM. A 72 h longer exposure with 5 or 10 mM metformin significantly reduced the proliferating cell population. An amount of 1 mM showed no inhibitory effect after 72 h compared to the control (Figure 2F).

### 3.3. Quantification of Antioxidant Effect, Apoptosis, and Proliferation

**Figure 3:**
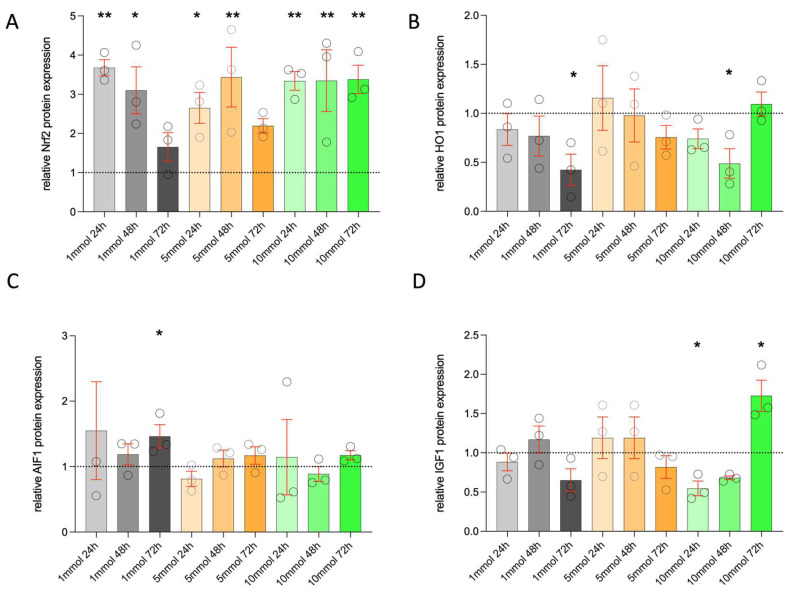
ELISA quantification of Nrf2 (**A**), HO-1 (**B**), AIF1 (**C**), and IGF-1 (**D**) of stimulated hMGECs. The raw data were normalized relative to the expression of the control (relative to control = 1, above 1 mean upregulated, below 1 means downregulated), statistic is the mean +/− SEM relative to the mean of the control (n = 3); * *p* < 0.05, ** *p* < 0.01 One-Way ANOVA (Kruskal–Wallis with Dunn’s test (uncorrected)).

Nrf2: With all metformin concentrations, the Nrf2 levels were increased compared to the control. Nrf2 showed different effects at different concentrations. After stimulation with 1 mM, Nrf2 increased the most at 24 h and declined over 48 and 72 h compared to levels at 24 h, though Nrf2 levels were more than those of the control at all time points (Figure 3A). After the 5 mM treatment, the expression peaked at 48 h and varied from 24 h to 72 h. With the 10 mM treatment, the increased levels of Nrf2 were maintained from 24 h to 72 h.

HO-1: The concentration of the antioxidant HO-1 was less than that of the control with all concentrations of metformin, except non-significant higher levels at 24 h (5 mM) and 72 h (10 mM). A significant decrease in HO-1 levels was observed with 72 h of 1 mM and 24 h of 10 mM metformin (Figure 3B).

AIF1: The expression of AIF1 was not significantly regulated at all time points and at different metformin concentrations, with the exception of 1 mM (72 h). The statistical significance of AIF1 was observed after 72 h of metformin stimulation with 1 mM concentrations (Figure 3C).

IGF-1: The concentration of IGF-1 showed a variable response with time and concentrations. IGF-1 levels increased with prolonged hours of stimulation, i.e., 48 h of 1 mM and 5 mM, but declined at 72 h of 1 mM and 5 mM exposure. With the 5 mM concentration, the levels were increased more than those of the control at 24 and 48 h but reduced after 72 h of stimulation, though these changes were insignificant (Figure 3D). Stimulation with 10 mM significantly reduced IGF-1 expression at 24 h and 48 h compared to the control, except for 10 mM at 72 h (Figure 3D). After 72 h of exposure to 10 mM, IGF-1 expression was significantly increased.

### 3.4. Expression of Stress Markers

**Figure 4:**
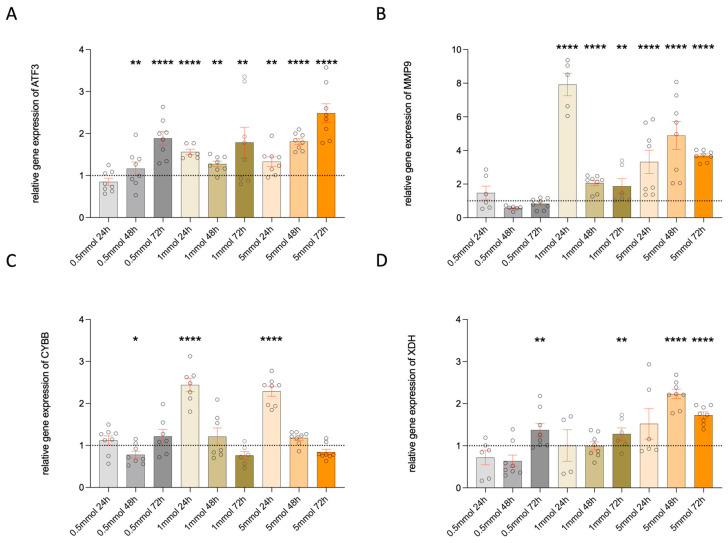
Expression of cell and oxidative stress marker of ATF3 (**A**), MMP9 (**B**), CYBB (**C**) and XDH (**D**)**.** Diagrams showing the differences in gene expression in hMGEC (n = 4). The data display the mean ± SEM from n = 4 per group. The raw data were normalized relative to the expression of the control (relative to control = 1, above 1 mean upregulated, below 1 means downregulated), * *p* < 0.05, ** *p* < 0.01, **** *p* < 0.0001 One-Way ANOVA (Kruskal–Wallis with Dunn’s test (uncorrected)).

To assess the influence of metformin on cell stress, we examined the expression of stress markers (matrix metallopeptidase 9, activating transcription factor 3) and oxidative stress markers (CYBB or NADPH oxidase 2 (NOX2), xanthine dehydrogenase) in hMGEC cells after metformin stimulation (Figure 4). To see if even a very low concentration has an impact on cell stress, another incubation with 0.5 mM was added. Stimulation with 10 mM for 72 h was not useful in the experiment, as the TEM analyses showed that we had a high number of cells with beginning and advanced cell death. All markers showed an increase with all metformin concentrations and time. A significant increase in MMP9, ATF3, CYBB, and XDH stress markers in metformin-treated cells indicated strong oxidative and cell stress.

## 4. Discussion

Metformin increases lipid production in hMGECs (at least in the tested cell line) and has a differentiating effect on the proliferating cells of hMGECs. Our results support the hypothesis that metformin intake would increase lipid production, which could increase the meibum volume. In addition to the increase in lipid bodies, keratin filament bundles can also be observed (Figure 1). Epithelial cells are abundant in keratin filament bundles, which form complex cytoskeletal networks crucial for cell type-specific functions. These keratin filaments support processes such as cell adhesion, migration, differentiation, and metabolism [19,20]. In sebocytes similar to HMGECs, targeted stimulation with IGF-1 can stimulate lipid production [21]. The effects of metformin were not observed via the regulation of IGF-1 levels in the current study because IGF-1 was not significantly increased. Ding et al. also showed no clear proliferative effect from the insulin-induced dose-dependent stimulation of hMGECs [22]. As hMGECs are specialized secretory epithelial cells that produce a lipid-rich secretion, further effects are suspected, even if they are thought to be similar to sebocytes. Our TEM analyses have shown that stimulation with metformin, especially at 10 mM for 48 h and 72 h, induces morphological cell death (Supplementary Figure S1). Real-time analyses also showed that metformin treatment induces oxidative stress (Figure 4). IGF-1 is a hormone that regulates key life history traits and influences aging. High IGF-1 levels are linked to increased mortality and may cause oxidative stress [23,24]. It is assumed that cell stress leads to a significant increase in IGF-1 at 10 mM and 72 h in the hMGECs. The relationship between metformin and IGF-1 requires further investigation, as the current study did not test IGF-1 pathway blocking.

Diabetes mellitus affects the ocular surface through keratopathy and dry eye disease (DED) [10]. The proposed mechanisms of DED in DM are dysregulation of the autonomic nervous system (an overactive sympathetic nervous system), abnormal mitochondrial metabolism in the lacrimal gland, corneal nerve degeneration, and hyperglycemia-induced glandular dysfunction [10,11,12,13,14]. There is a higher MGD prevalence in DM than in non-diabetics [25,26]. There is consensus on the meibomian glands’ morphological changes in many studies on DM patients, whereas non-uniformity is noted in tear film parameters. Metformin, being a hypoglycemic drug, is commonly used for treating DM. The effects of hyperglycemia and metformin use on meibum and meibomian glands in DM individuals with or without DED will be useful. The current study investigated the effects of different concentrations of Metformin on meibomian gland epithelial cells. The proliferating effects were dependent on the time and dose of the metformin. Metformin had cytotoxic effects in hMGEC cultures at concentrations of 10 mM at 48 and 72 h (Supplementary Figure S1), resulting in a decrease in cell proliferation (Figure 2). Whether the necroptosis and apoptosis signaling pathway was activated is not known. From the literature, necroptosis can be inferred [27], and our assumption is confirmed by the fact that AIF1 was not clearly regulated. The previously described effect that metformin induces the apoptosis of lung cancer cells could not be demonstrated in hMGEC or confirmed by the AIF1 factor [28]. This is a limitation in our project, and further studies are needed to show this.

The mechanisms of action of metformin on hMGECs are unclear. Metformin is thought to induce abnormal mitochondrial metabolism in the lacrimal gland [10], while we found it to have the opposite effect on cultured hMGECs. We found an increase in the expression of stress markers (matrix metallopeptidase 9, activating transcription factor 3) and oxidative stress markers (CYBB or NADPH oxidase 2, xanthine dehydrogenase) after metformin treatment. Oxidative stress leads to an increase in reactive oxygen species, which leads to a dysregulation of the inflammatory response. This is key to the onset and progression of diabetic neurodegenerative diseases and leads to the activation of proinflammatory cytokines and chemokines [29,30,31]. Diabetes is usually associated with an increased production of free radicals and/or a weakening of antioxidant defense mechanisms. Hyperglycemia in diabetics also affects the meibomian glands. Four months of hyperglycemia in a diabetic rat model was associated with acini dropout and an increased expression of inflammatory factors within meibomian glands. At cellular levels, there was a reduction in oxidative stress-related factors Nrf2 and HO-1, an increase in apoptotic cells, and a downregulation of phospho-AMP-activated protein kinase (AMPK) within meibomian glands [12]. Metformin in this diabetic rat model improved the stress levels within meibomian glands, along with a reduction in inflammation. AMPK levels were restored with metformin treatment. However, the needed dose (700 mg/kg daily gavage) was higher than for other tissues like skin and lens. The lipid production and anti-oxidant Nrf2 and HO-1 levels also improved with metformin in the rat model. The current study also showed increased Nrf2 levels with all metformin concentrations and time. Another study showed the antioxidant effects of low doses of metformin on epithelial cells of the meibomian glands through the multifunctional regulator nuclear factor erythroid 2-related factor (Nrf2), which is a cytoprotective factor that regulates the expression of genes coding for antioxidant, anti-inflammatory, and detoxifying proteins [32]. However, a downstream regulation of another antioxidant protein, HO-1, could not be established. The Nrf2 levels in hMGECs increased, while HO-1 decreased significantly. This suggests that metformin has different effects on, for example, normal mitochondrial metabolism. An increased Nrf2 activity defends against mitochondrial toxins and promotes lipid synthesis [33]. HO-1 protects cells by reducing oxidative stress and inflammation and maintaining mitochondrial integrity, thereby promoting cell survival [34]. The downregulation of HO-1 in the current study suggests that this protective function is impaired, as there was an increase in all cell stress markers in stimulated cells. Another possibility could be a failure of Nrf2 to activate the downstream pathway of HO-1 in hMGECs. In the diabetic rat model, the relative protein concentrations of Nrf2 and HO-1 were downregulated in tissue and serum [35]. In another study, metformin was shown to enhance the Nrf2/HO-1 signaling pathway [36]. hMGECs are a new cell line, and it can only be speculated as to why HO-1 expression decreases in hMGECs. The research group led by Yu et al. has shown that metformin inhibits HO-1 expression in A549 cells [37]. Further studies on hMGECS will lead to a more comprehensive understanding of metformin effects on the Nrf2/HO-1 signaling pathway.

The current study results indicate that the regulation of metformin effects on cell stress is dose and duration dependent. In a diabetic mouse, the salivary glands accumulated two-fold metformin compared to plasma levels [38]. Distribution studies of metformin within the human body have demonstrated that when 2 g/day of metformin is consumed, 6 mM is excreted daily via the kidneys, and the colon has concentrations of up to 40 mM metformin [39]. It is unknown how much metformin reaches the meibomian glands in animal or human studies. Based on the results of the current study, higher levels are not beneficial for meibomian glands.

Metformin reduces lipid peroxidation in the skin and lens of diabetic mice and improves glutathione levels [40]. Metformin is being explored as a treatment option for Sjogren’s syndrome [41,42]. Its anti-inflammatory effects were observed in reducing helper T cell, IL-6, and IL-17 levels and increasing regulatory T cells within the salivary glands of the NOD mouse model [42]. The anti-inflammatory effects were exerted via AMPK activation secondary to the inhibition of mitochondrial respiratory chain complex I and subsequent changes in the AMP to ATP ratio [41]. AMPK activation inhibits the mammalian target of rapamycin (mTOR), which regulates T-cell differentiation. However, the anti-inflammatory effect would be relevant in diabetics, where increased inflammation was observed in the meibomian glands of diabetic mice models. The current study did not look at the expression of inflammatory cells or cytokines *in vitro*. However, their levels are elevated in diabetic rat models that are reversed with metformin. Studying hMGECs exposed to hyperglycemia or IL-1β would offer a better insight into the impact of metformin on the meibomian glands of diabetics.

## 5. Conclusions

Our results suggest that metformin affects the cellular physiology of meibomian gland epithelial cells and induces cell stress in a dose- and time-dependent manner, causing changes in their morphology.

The changes in meibum components after metformin treatment will be of interest for future studies, and the lipid increase at low doses is of high clinical relevance for the treatment of lipid-dependent DED. As even low doses of metformin trigger increased cell stress, it is all the more important to understand how the drug affects other organ systems in order to optimize patient treatment in the future.

## Figures and Tables

**Table 1:** List of used TaqMan^®^ gene expression assays.

TaqMan™ Genexpressionsassay (FAM) ATF3 (Hs00231069_m1)	Thermo Fisher
TaqMan™ Genexpressionsassay (FAM) GAPDH (Hs02786624_g1)	Thermo Fisher
TaqMan™ Genexpressionsassay (FAM) MMP9 (Hs00957562_m1)	Thermo Fisher
TaqMan™ Genexpressionsassay (FAM) CYBB = NOX2 (Hs00166163_m1)	Thermo Fisher
TaqMan™ Genexpressionsassay (FAM) XDH (Hs00166010_m1)	Thermo Fisher

**Table 2:** List of real-time RT-PCR primer sequences.

NAME	FORWARD	REVERSE	SIZE
hu CYP1A1 RT	ccc aac cct tcc ctg aat g	ttc tcc tga cag tgc tca atc	146 bp
hu PPARγ RT	gac agg aaa gac aac aga caa atc	ggg gtg atg tgt ttg aac ttg	96 bp
hu 18S RT	gga gcc tga gaa acg gct a	tcg gga gtg ggt aat ttg c	64 bp

## Data Availability

The original contributions presented in the study are included in the article/Supplementary Material, further inquiries can be directed to the corresponding author.

## Supplementary Materials

The following supporting information can be downloaded at: https://www.mdpi.com/article/10.3390/life14121682/s1, Figure S1. Morphology: (A) LowMAG (×600) electron micrograph images of a stimulated hMGEC with 10 mM metformin at 24–72 h. After 72 h with 10 mM metformin, most cells show low electron density of the cytoplasm, and organelles are completely destroyed (red X). (B) Magnification of an hMGEC stimulated by 10 mM for 48 h and 72 h. Red points = lipid bodies; green points = lipid bodies associated vacuoles; green arrowheads = phagocytotic bodies; n = nucleolus; blue stars = keratin filament. Scale bar = 5 µm.
