# Supplementary material for: Effect of Metformin on Meibomian Gland Epithelial Cells: Implications in Aging and Diabetic Dry Eye Disease
Source: Life (Basel). 2024 Dec 18;14(12):1682. doi: 10.3390/life14121682 (PMC11679316; doi:10.3390/life14121682)
Supplement: Supplementary file 1 [file life-14-01682-s001.zip › life-3299899-supplementary.pdf]

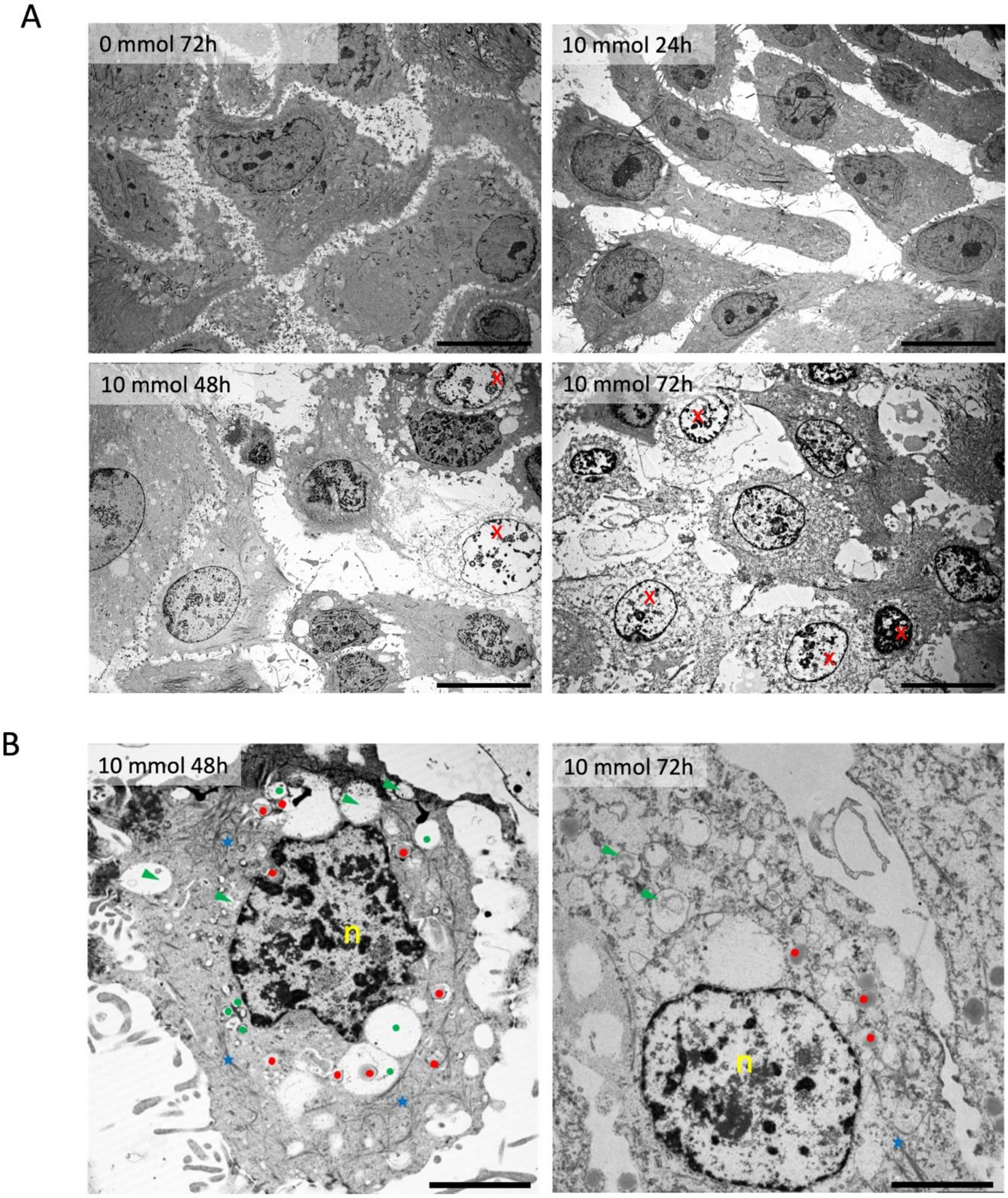

Figure S1. Morphology: (A) LowMAG (×600) electron micrograph images of a stimulated hMGEC with 10 mM metformin at 24–72 h. After 72 h with 10 mM metformin, most cells show low electron density of the cytoplasm, and organelles are completely destroyed (red X). (B) Magnification of an hMGEC stimulated by 10 mM for 48 h and 72 h. Red points = lipid bodies; green points = lipid bodies associated vacuoles; green arrowheads = phagocytotic bodies; n = nucleolus; blue stars = keratin filament. Scale bar = 5 µm
